# Using pentosidine and hydroxyproline to predict age and sex in an avian species

**DOI:** 10.1002/ece3.3388

**Published:** 2017-09-25

**Authors:** Brian S. Dorr, Randal S. Stahl, Katie C. Hanson‐Dorr, Carol A. Furcolow

**Affiliations:** ^1^ US Department of Agriculture Wildlife Services National Wildlife Research Center Mississippi Field Station Starkville MS USA; ^2^ US Department of Agriculture Wildlife Services National Wildlife Research Center Fort Collins CO USA

**Keywords:** advanced glycation end products, aging, cross‐linking theory, Double‐crested Cormorants, error‐damage theory, *Phalacrocorax auritus*

## Abstract

All living organisms are subject to senescence accompanied by progressive and irreversible physiological changes. The error damage and cross‐linking theories suggest that cells and tissues are damaged by an accumulation of cross‐linked proteins, slowing down bodily processes and resulting in aging. A major category of these cross‐linked proteins are compounds called advanced glycation end products (AGEs). We investigated the relationship between accumulation of the AGE, pentosidine (Ps), and hydroxyproline (HYP) a post‐translationally modified amino acid, with age, sex, and breeding status (breeder/nonbreeder) from skin samples of known age (i.e., banded as fledglings), free‐ranging Double‐crested Cormorants (*Phalacrocorax auritus,* Lesson 1831). We developed multivariate models and evaluated the predictive capability of our models for determining age and breeding versus nonbreeding birds. We found significant relationships with Ps and HYP concentration and age, and Ps concentration and sex. Based on our two‐class model using Ps and HYP as explanatory variables, we were able to accurately determine whether a cormorant was a breeder or nonbreeder in 83.5% of modeled classifications. Our data indicate that Ps and HYP concentrations can be used to determine breeding status of cormorants and potentially age of cormorants although sex‐specific models may be necessary. Although the accumulation of Ps explained the greatest amount of variance in breeding status and age, importantly, Ps covaried with HYP and combined improved prediction of these demographics in cormorants. Our data support the error damage and cross‐linking theories of aging. Both Ps and HYP increase predictably in cormorants and are predictive of age and breeding status. Given the ubiquity of these biomarkers across taxa, their use in estimating demographic characteristics of animals could provide a powerful tool in animal ecology, conservation, and management.

## 1 INTRODUCTION

All living organisms are subject to senescence, and in the process, there are progressive and irreversible physiological changes that occur. There are many theories regarding why organisms age, one being the damage or error theory (Jin, 2010). One proposed mechanism of the error‐damage theory includes the cross‐linking theory (Davidovic et al., 2010; Jin, 2010). This theory suggests that cells and tissues are damaged by an accumulation of cross‐linked proteins, slowing down bodily processes and resulting in aging (Bjorksten, 1968; Bjorksten & Tenhu, 1990; Jin, 2010). A major category of these cross‐linked proteins are compounds called advanced glycation end products (AGEs) (Cerami, Vlassara, & Brownlee, 1987). These AGEs are irreversible, stable, and accumulate in tissues (Fallon, Radke, & Klandorf, 2006). One type of AGE, pentosidine (Ps), is naturally occurring, fluorescent, and has found use as a biomarker related to oxidative stress, age‐related diseases, and aging itself (Rosenberg & Clark, 2012).

Accumulation of Ps with age has been documented in numerous mammal and avian species (Chaney, Blemings, Bonner, & Klandorf, 2003; Dammann, Sell, Begall, Strauch, & Monnier, 2012; Fallon, Cochrane, Dorr, & Klandorf, 2006; Fallon, Radke, et al., 2006; Iqbal, Probert, Al‐humadi, & Klandorf, 1999; Sell et al., 1996). Comparison of Ps accumulation in mammals and birds has demonstrated that Ps accumulates at different rates in different species, and longer‐lived species accumulate Ps at slower rates than shorter‐lived species (Fallon, Cochrane, et al., 2006; Fallon, Radke, et al., 2006; Sell et al., 1996). Cooey et al. (2010) found that relatively small skin samples (6 mm biopsy) can be used to quantify Ps concentrations, allowing for nondestructive sampling of wildlife. The ubiquity of Ps, its accumulation with age, the inverse relationship between accumulation rate of Ps and longevity, and the ability to nondestructively sample suggest that Ps is suitable to use as a biomarker to age a wide variety of organisms (Chaney et al., 2003; Fallon, Cochrane, et al., 2006; Fallon, Radke, et al., 2006).

While the basic relationship between accumulation of Ps and age and longevity has been demonstrated (Baynes, 2001; Dammann et al., 2012), the nature and utility of that relationship is less certain. Chaney et al. (2003) found that Ps accumulation was linearly correlated to age in a variety of wild bird species, whereas Fallon, Cochrane, et al. (2006), found a linear relationship in Double‐crested Cormorants (*Phalacrocorax auritus*, Lesson 1831; hereafter cormorant), and a nonlinear relationship in Ruffed Grouse (*Bonasa umbellus,* Linnaeus 1766). Furthermore, the slope of the regressions for each species were different, with the shorter‐lived Ruffed Grouse accumulating Ps much more rapidly than the cormorant (Fallon, Cochrane, et al., 2006).

Multiple factors can affect the accumulation of Ps with age within a species. Accumulation of Ps is related to oxidative stress, and therefore, endogenous or exogenous factors causing stress in an individual can potentially affect Ps accumulation (Costantini, Fanfani, & Dell'Omo, 2008; Rosenberg & Clark, 2012). Dietary restriction combined with an oxidative inhibitor (i.e., aminoguanidine) has been shown to reduce accumulation of Ps (Iqbal et al., 1999; Novelli, Masiello, Bombara, & Bergamini, 1998; Sell et al., 2003). Life‐history events, such as breeding, or physiological traits, such as sex, could also affect rates of accumulation of Ps (Dammann et al., 2012). In fact, some researchers have found conflicting information with regard to the relationship of Ps with age (Rattiste et al., 2015) and consequently its utility as a biomarker for aging.

In addition to inherent individual variability in accumulation of Ps, there are a number of other AGEs and related compounds that may covary with Ps and age. Concentration of Ps is typically reported in terms of the collagen content of the tissue which is calculated using a fixed ratio of hydroxyproline (HYP), a major component of the protein collagen. In typical assays of Ps, the amount of HYP is not considered to vary with age. However, HYP may in fact vary with age and covary with Ps concentration suggesting that a multivariate measure of association of these compounds and age could improve age prediction estimates.

**Figure 1:**
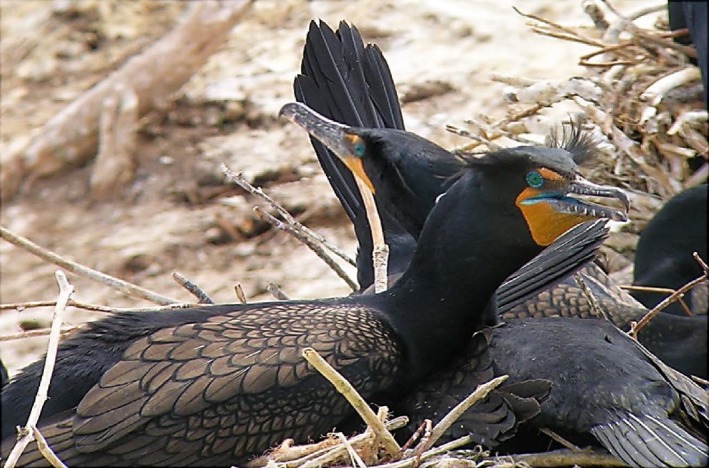
Nesting Double‐crested Cormorants (*Phalacrocorax auritus,* Lesson 1831). Photograph by D. T. King, US Department of Agriculture, Wildlife Services, National Wildlife Research Center

In this study, we investigated the relationship between accumulation of Ps and HYP with age, sex, and breeding status (breeder/nonbreeder) from skin samples of known age (banded as fledglings), free‐ranging Double‐crested Cormorants which are a North American colonial waterbird (Figure 1). Given the error damage theory of aging, we predicted that Ps would vary directly with age and vary by sex. We also predicted that HYP would covary with Ps and age and sex. We used resulting models to determine whether we could detect differences in Ps concentration between breeders and nonbreeders assuming that breeding would be an endogenous stress factor influencing Ps concentration. Lastly, we evaluated the predictive capability of our models for determining breeding versus nonbreeding birds.

## 2 MATERIALS AND METHODS

### 2.1 Skin sample collection

Skin samples were obtained from known age cormorant carcasses, 1–5 years old at time of collection, salvaged from United States Department of Agriculture, Wildlife Services population management activities. Four birds of each sex were sampled at each year except for the 4–year‐old category where samples were collected from two females and three males; and in the 5‐year‐old category where three females were sampled. Feathers were removed from the upper breast region, and a 6‐cm^2^ skin sample was dissected and removed. Samples were stored in water and frozen at −12°C until prepared for analysis. A small ~40 mg subsample was isolated from the skin tissue and scraped with a scalpel to remove any remaining feathers and subcutaneous adipose tissue.

### 2.2 Sample hydrolysis

Skin subsamples were extracted in 2 ml of 2:1 chloroform/methanol (MeOH) to remove the lipid fraction (Folch, Lees, & Stanley, 1957; Hamilton, Hamilton, & Sewell, 1992). The solvent was decanted, and the remaining samples were dried under a mild stream of N_2_ at 40°C after rinsing with 2 ml of MeOH, then acid was hydrolyzed in 5 ml of 6 N HCl with a CEM Discover SPD microwave digester. Samples were digested in a 10‐ml quartz tube. Temperature was maintained at 185°C after ramping from ambient over 5 min. Samples were exposed to a maximum microwave energy of 300 W and digested at a maximum pressure of 200 psi. Samples were continuously stirred with a magnetic stir bar during digestion. The acid hydrolysate was dried under vacuum at 60°C with a Rotovap. The sample was reconstituted in 1.0 ml of 25% MeOH in water containing 0.1% heptafluorobutyric acid (HFBA).

### 2.3 Pentosidine determination

Pentosidine was determined in the hydrolysate using a high‐performance liquid chromatography (HPLC) method based on that of Bank et al. (1997) following centrifuge filtration of the extract using a Durapore PVDF 0.45‐μ filter (Millipore). Chromatography was performed using an Agilent 1100 LC with a fluorescence detector. A 5 μl sample was injected onto an Agilent XDB‐C8 3 × 150 mm, 3.5 μ column. Pentosidine was eluted in a gradient starting at 80/20% water/MeOH and ramping to 70/30% water/MeOH containing 0.1% HFBA at 4 min at a constant flow rate of 0.95 ml/min. The MeOH was ramped to 60% at 8.4 min and then to 100% at 10 min and held constant for 4 min to flush the column. The column was re‐equilibrated for 3 min prior to the next injection. The column was maintained at 60.0°C. Pentosidine was detected by fluorescence, exciting at 328 nm and monitoring for fluorescence emission at 378 nm. Pentosidine concentration was determined using an external standard curve covering the concentration range of 0–25 pmol/ml prepared from a primary standard acquired from Polypeptide (Strasbourg, FR). Pentosidine eluted at approximately 4.7 min under these chromatographic conditions. Pentosidine concentration is reported in pmol/mg collagen.

### 2.4 Collagen determination

Collagen content in the tissues was estimated by determining the concentration of HYP in the hydrolysate. Hydroxyproline determination was based on the secondary amine derivatization method of Hutson, Crawford, and Sorkness (2003). To summarize this method, primary amines are derivitized with o‐pthalaldehyde and then secondary amines are derivitized with fluorenylmethyloxycarbonyl chloride (FMOC). We adapted this method by diluting 0.05 ml of our extract with 0.2 ml of 0.25 M boric acid, pH 9.5, and 0.05 ml of a 200 ppm sarcosine internal standard in 0.70 ml of water. We follow the rest of the derivitization procedure as published. Concentrations were determined with an external standard curve over the range of 0–250 ppm HYP using a primary standard (Sigma‐Aldrich). Concentration values for μg HYP/mg lipid‐free sample are reported.

We injected 1 μl of the derivitized solution on a Luna C18 (2) 3 × 75 mm, 3 μ column and used a gradient separation with an Agilent 1100 HPLC with a fluorescence detector to quantify the amount of HYP present. The mobile phase started with a composition of 3% acetic acid (pH: 4.3)/acetonitrile 75/25% and was ramped to 25/75% from 2 to 10 min, postinjection at a flow rate of 0.3 ml/min. This composition was maintained for 3 min, after which the column was re‐equilibrated for 7 min prior to the next injection. The FMOC‐derivitized HYP and sarcosine were excited at 265 nm, and fluorescence emission was detected at 330 nm. Separation was performed at a column temperature of 40°C. Hydroxyproline eluted at approximately 7.7 min while sarcosine eluted at 10.3 min. Collagen content was estimated from the HYP concentration and was referenced to Peptan 5000 HD (Rousellot), a collagen calf skin material containing 11.5% HYP and reported as mg collagen.

### 2.5 Data analysis

Trends in the concentration of HYP (μg/mg skin) and Ps (pmol/mg collagen) in the samples were investigated using linear regression models, where the independent variable was age (months). The interaction of sex and age for each analyte was evaluated using a two‐way ANOVA where the grouping was by age (years). Due to incomplete block sample size for 4‐year‐old female birds, the 4‐year data were not included in this analysis. ANOVA statistical analysis was performed using “R.” A multivariate regression analysis was also performed looking at the effect of Sex, HYP, Ps, and the interaction of HYP:Ps on age predications. The models were compared using Akaike's information criterion (AIC_c_) to select the best model.

A two‐class model to evaluate the feasibility of differentiating immature birds from sexually mature birds was developed using two different statistical analysis. Cormorants were considered to be sexually mature by their third year (Dorr, Hatch, & Weseloh, 2014). The first analysis predicted age class based on multivariate regression models using Ps and HYP concentrations determined for birds of known age discussed above. The second analysis used a multivariate approach based on principal component (PCA) scores derived from the sex, HYP concentration, and the Ps concentration determined for each bird. The two‐class PCA‐based model used linear discriminant analysis (LDA) to predict membership in a class, while the regression‐based model predicted age class membership directly from a measured Ps concentration. Both approaches were evaluated by randomly selecting a subset of the data, used to test the model, and the remaining data were used to fit a model. For the regression models, three datasets were withheld for model prediction, across sex. In contrast, the PCA‐based model was fit by randomly selecting the PCA scores for 28 of the birds. The resulting model was then used to predict the class of the remaining eight birds. In both cases, this was repeated 100 times to assess the classification error rate for the respective model. These statistical analyses were performed using the package “chemometrics” in “R” as described by Varmuza and Filmozer (2009).

## 3 RESULTS

**Figure 2:**
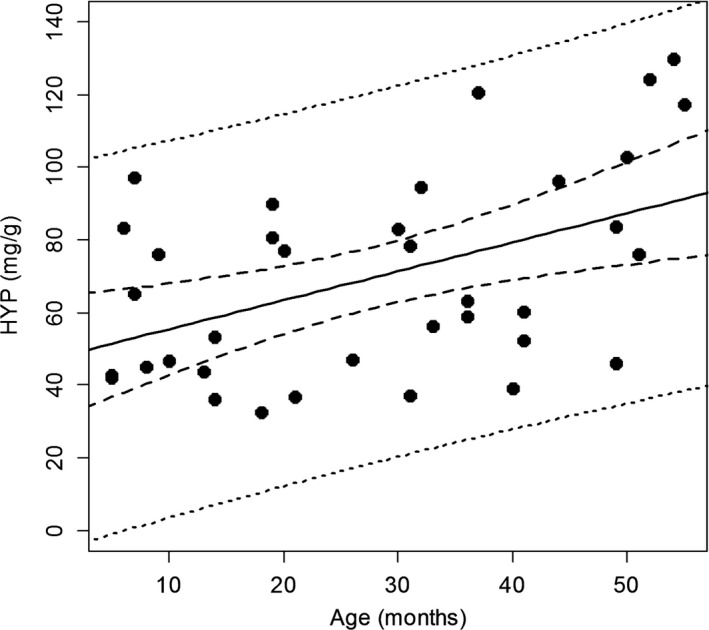
Hydroxyproline concentration by age for Double‐crested Cormorants (*Phalacrocorax auritus*) determined from skin tissue samples of known age wild cormorants banded as fledglings. Solid line is the mean trend, heavy dotted line is the 95% CI, and the light dotted line is 2 *SD* from the mean

**Figure 3:**
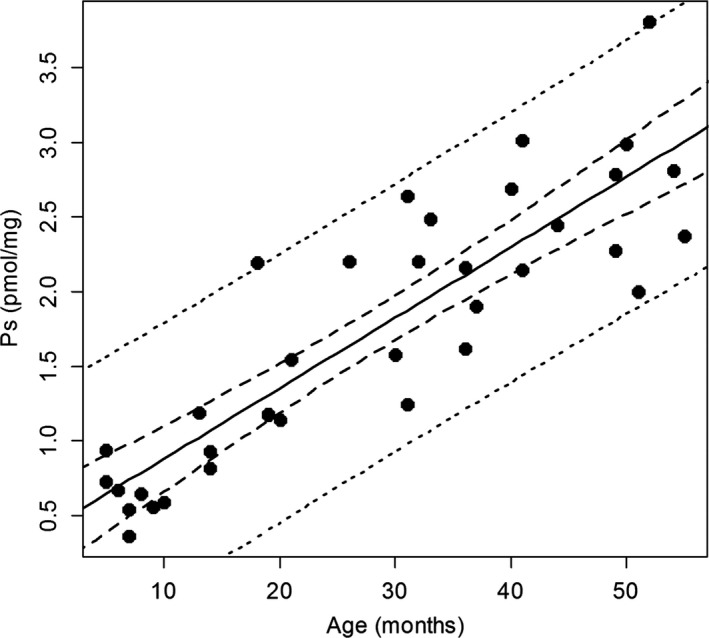
Pentosidine regression by age for Double‐crested Cormorants (*Phalacrocorax auritus*) determined from skin tissue samples of known age wild cormorants banded as fledglings. Solid line is the mean trend, heavy dotted line is the 95% CI, and the light dotted line is 2 *SD* from the mean

The mean concentrations for HYP and Ps determined for the samples are presented in Table 1, grouped by sex and age class. The measured values for HYP and Ps concentrations for each cormorant by age are plotted in Figures 2 and 3, respectively. The equation describing the change in HYP concentration is as follows: concentration (mg/g) = 0.80 × Age (months) + 47.4. Both the slope and the intercept are significant at the α = 0.05 level. The equation describing the relationship between Ps concentration and age is as follows: concentration (pmol/mg collagen) = 0.047 × Age (months) + 0.41. Both the intercept and the slope are significant at the α = 0.05 level. The two‐way ANOVA results indicate that for both Ps and HYP concentrations, there is a significant relationship with age (*F*_3_ = 12.52, *p *<* *.0001 and *F*_3_ = 4.43, *p = *.012, respectively). For Ps, there is also a significant relationship with sex (*F*_1_ = 6.26, *p = *.019). The equations describing the relationship between age and Ps concentration for the males and females sampled, respectively, are as follows: concentration in male sample (pmol/mg collagen) = 0.047 × Age (months) + 0.45 and, concentration in female sample (pmol/mg collagen) = 0.046 × Age (months) + 0.38. In both cases, the slope is significant at the α = 0.05 level. In neither case was the intercept significant at the α = 0.05 level.

**Table 1:** Mean hydroxyproline (HYP) and pentosidine (Ps) concentrations observed in skin samples of male and female Double‐crested Cormorants (*Phalacrocorax auritus*) from one to 5 years of age

Sex	Age (years)	*n*	HYP (mg/g)	Ps (pmol/mg)
Male	1	4	59.4 ± 18.8	0.68 ± 0.18
	2	4	64.3 ± 23.8	1.20 ± 0.26
	3	4	61.6 ± 23.9	2.37 ± 0.23
	4	3	69.5 ± 23.9	2.48 ± 0.37
	5	4	112.0 ± 24.4	2.75 ± 0.78
Female	1	4	65.2 ± 26.2	0.57 ± 0.15
	2	4	48.3 ± 22.1	1.34 ± 0.59
	3	4	67.9 ± 16.2	1.66 ± 0.40
	4^a^	2	79.9	2.30
	5	3	77.4 ± 28.7	2.68 ± 0.37

Values are means ± 1 standard deviation.

a Standard deviations are not calculated because *n *<* *3.

The multivariate models for the effects of Sex, HYP, Ps and the interaction between HYP:Ps are presented in Table 2. The model with the lowest AIC_c_ is the HYP + Ps model. The ∆AIC_c_ value was set to zero for this model, and all other models were compared to it. The top three models ranked by AIC_c_ weight scores are as follows: HYP + Ps; HYP + Ps + HYP:Ps, and the Sex + HYP + Ps model. There is very little difference in the ΔAIC_c_ values between these three models given the magnitude of the AIC values.

**Table 2:** Model selection results for the regression models evaluating the relationship between HYP, Ps, sex, and age (months) in the Double‐crested Cormorants (*Phalacrocorax auritus*)

Model	*K*	Adj. *R* ^2^	AIC_c_	∆AIC_c_	AIC_c_ weights
Sex + Ps	4	0.75	259.6	10.7	0.0026
Sex + HYP	4	0.18	302.5	53.6	1.27e‐12
Sex + HYP + Ps	5	0.81	251.3	2.4	0.17
Sex + HYP + Ps + HYP:Ps	6	0.81	253.4	4.5	0.058
HYP + Ps	4	0.81	248.9	0	0.55
HYP + Ps + HYP:Ps	5	0.81	250.8	1.9	0.21
HYP	3	0.20	300.2	51.3	4.0e‐12
Ps	3	0.76	257.1	8.2	0.0091
Intercept	2	—	306.8	57.9	1.47e‐13

*K* is the number of parameters in the model. Adj. *R* ^2^ is the *R* ^2^ value adjusted for the number of parameters in the model and accounts for the amount of variation explained by the model. AIC_c_ is Akaike's information criterion adjusted for small sample size. ∆AIC_c_ are the calculated differences in the AIC_c_ of the best model and the selected model. The AIC_c_ weights reflect the relative support for a selected model.

In the first analysis, the two‐class regression‐based models had different misclassification rates for the two sexes. Predicting the age class of three birds from models built from the remaining data for the males across 100 iterations of the model resulted in immature birds being classified as mature birds 5.3% of the time. Mature male birds were never misclassified as immature birds using the data available to us. Female misclassification rates were higher for both classes with immature birds being classified as mature birds 13.3% of the time and mature birds being classified as immature 9.3% of the time. The total misclassification rate for the female birds was 22.7%.

**Figure 4:**
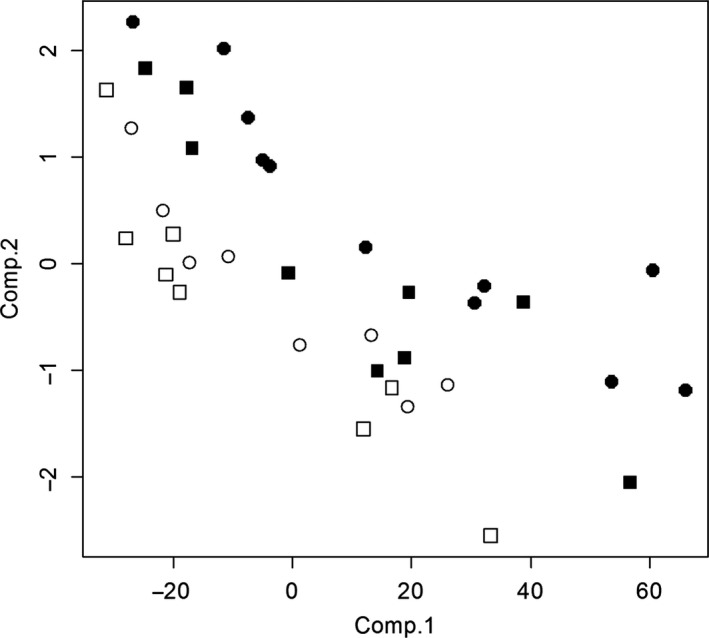
Principle component analysis score plot for models predictive of nonbreeding (1–2 years old) Double‐crested Cormorants (*Phalacrocorax auritus*) and adult, breeding (≥3 years old) cormorants. Symbols represent: Immature females (open squares), immature males (open circles), mature females (closed squares), and mature males (closed circles)

In the second analysis, the LDA classification model using the component score for each bird plotted in Figure 4 and iterated 100 times successfully predicted whether a cormorant was immature or mature 83.5% of the time. Only 6.0% of the false classifications were for immature cormorants being classified as mature. The remaining 8.5% of false misclassifications were for mature birds being classified as immature birds. This multivariate approach improves age‐based classification estimates over the commonly used regression estimates, particularly for the female cormorants.

## 4 DISCUSSION

We found significant relationships between both Ps and HYP concentration and age, and Ps concentration and sex. Our findings are consistent with the error damage theory of aging and the role of cross‐linked proteins as a possible mechanism for aging. Further, our data indicate that Ps concentrations can be used to determine breeding status of cormorants, and potentially age, although sex‐specific models may be necessary.

As with Fallon, Cochrane, et al. (2006), we found a significant and direct relationship with concentration of Ps and age in cormorants. Also like Fallon, Cochrane, et al. (2006), the relationship we describe for cormorants appears to be linear rather than nonlinear. Although, Fallon, Cochrane, et al. (2006), sampled across a greater range of age classes (1–15), their sample was largely bi‐modal (<4 years and >8 years in age). We evaluated multiple samples within each age class (1–5 years of age), thus providing a more rigorous assessment of the relationship between Ps and age across these age classes. Regardless, our findings largely confirm those of Fallon, Cochrane, et al. (2006), and indicate that Ps increases in vivo with age in cormorants. We also found that HYP increased with age, although at a different rate and with greater individual variation than Ps (Figures 1 and 2); however, unlike Ps, we did not find a significant relationship between HYP and sex.

Based on our two‐class PCA multivariate model using Ps and HYP as explanatory variables, we were able to accurately determine whether a cormorant was a breeder or nonbreeder in 83.5% of modeled classifications. We note that although most cormorants breed at 3 years of age, some do breed earlier (Dorr et al., 2014), which may have reduced the accuracy of this model. In addition, our sample size is small relative to parameters evaluated, particularly for some age classes. Regardless, both factors combined explained a significant amount of variation in age and improved accuracy of models predictive of age. These results demonstrate the feasibility of distinguishing nonbreeding (≤2 years old) cormorants from breeding (≥3 years old) cormorants (Dorr et al., 2014) using HYP and Ps concentrations from a small skin sample. These findings also suggest that multivariate measures of Ps and HYP, and possibly other AGEs, could improve these techniques as biomarkers of chronological age in living organisms. To the best of our knowledge, no multivariate model of AGEs has been used to predict demographics of a vertebrate species. Further, we are not aware of a model using AGEs whose predictive accuracy has been evaluated through resampling modeling as has been performed in this study.

Our findings are consistent with and supported by the underlying error damage theory of aging (Bjorksten, 1968; Bjorksten & Tenhu, 1990; Jin, 2010). Given this theory, we would predict that Ps would accumulate with age of an organism as demonstrated with cormorants in this study. Importantly, this accumulation of Ps covaried with HYP and combined was used to predict breeding status and age of cormorants with a relatively high degree of accuracy. However, because accumulation of Ps is affected by endogenous and exogenous factors (Costantini et al., 2008; Davidovic et al., 2010; Rosenberg & Clark, 2012), there is substantial variation in age estimates and multiple methods may be needed to improve model performance in predicting chronological age of organisms using Ps accumulation alone. Given that there are many types of AGEs that may be used in this capacity (Davidovic et al., 2010), a multivariate model may be one approach for improving methods for estimating age in organisms that are more accurate and valuable for assessing species demographics.

Developing a relatively rapid, nonlethal means for estimating age or breeding status in organisms could prove highly valuable to those involved with conservation and management of wildlife. Many species such as the Double‐crested Cormorant are monotypic as adults making determination of their population demographics a difficult and time‐consuming process usually involving the marking (banding) and recapture of many individuals over many years (Chaney et al., 2003; Fallon, Cochrane, et al., 2006; Fallon, Radke, et al., 2006). Given the ubiquity of Ps and AGEs across taxa, and their characteristic of accumulation in living tissue over time, further investigation and refinement of their use in aging wildlife could provide a powerful tool in animal ecology, conservation, and management.

## AUTHORS’ CONTRIBUTIONS

BSD and RSS conceived the ideas and designed methodology; RSS, KCH‐D, and CF collected the data; RSS analyzed the data; BSD and RSS led the writing of the manuscript. All authors contributed critically to the drafts and gave final approval for publication.

## DATA ACCESSIBILITY

Data are archived at the USDA, Wildlife Services, National Wildlife Research Center, Fort Collins, Colorado, USA, www.aphis.usda.gov/aphis/ourfocus/wildlifedamage/programs/nwrc/sa_about/ct_contacts_nwrc.

## CONFLICTS OF INTEREST

None declared.
